# lncRNA SNHG1 regulates odontogenic differentiation of human dental pulp stem cells via miR-328-3p/Wnt/β-catenin pathway

**DOI:** 10.1186/s13287-022-02979-w

**Published:** 2022-07-15

**Authors:** Tingting Fu, Yiran Liu, Xin Huang, Yan Guo, Jiaping Shen, Hong Shen

**Affiliations:** Department of Pediatric and Preventive Dentistry, Jiangsu Key Laboratory of Oral Diseases, Affiliated Hospital of Stomatology, Nanjing Medical University, Nanjing, 210029 China

**Keywords:** lncRNA SNHG1, Odontogenic differentiation, hDPSCs, miR-328-3p, Wnt/β-catenin

## Abstract

**Background:**

Elucidating the mechanism of odontogenic differentiation of human dental pulp stem cells (hDPSCs) is the key to in-depth mastery and development of regenerative endodontic procedures (REPs). In odontogenic differentiation, lncRNAs have a regulatory role. The goal of this research is to determine the involvement of short nucleolar RNA host gene 1 (SNHG1) in hDPSCs’ odontogenic differentiation and the mechanism that underpins it.

**Methods:**

hDPSCs were isolated from the dental pulp tissue of healthy immature permanent teeth. Follow-up experiments were performed when the third generation of primary cells were transfected. The proliferation ability was measured by CCK-8. The biological effects of SNHG1 and miR-328-3p were determined by real-time quantitative polymerase chain reaction (qRT-PCR), western blot (WB), alkaline phosphatase (ALP) staining and activity, alizarin red S staining (ARS) and quantification, and immunofluorescence staining. The binding of SNHG1 and miR-328-3p was confirmed using a dual-luciferase reporter assay. qRT-PCR and WB were used to determine whether the canonical Wnt/β-catenin pathway was activated.

**Results:**

On the 0th, 3rd, and 7th days of odontogenic differentiation of hDPSCs, SNHG1 showed a gradual up-regulation trend. SNHG1 overexpression enhanced the mRNA and protein expression of dentin sialophosphoprotein (DSPP), dentine matrix protein 1 (DMP-1) and ALP. We found that SNHG1 could bind to miR-328-3p. miR-328-3p inhibited the odontogenic differentiation of hDPSCs. Therefore, miR-328-3p mimics rescued the effect of SNHG1 overexpression on promoting odontogenic differentiation. In addition, SNHG1 inhibited Wnt/β-catenin pathway via miR-328-3p in odontogenic differentiation of hDPSCs.

**Conclusion:**

lncRNA SNHG1 inhibits Wnt/β-catenin pathway through miR-328-3p and then promotes the odontogenic differentiation of hDPSCs.

**Supplementary Information:**

The online version contains supplementary material available at 10.1186/s13287-022-02979-w.

## Introduction

Recently, regenerative endodontic procedures (REPs) have become an important method in treating young permanent teeth with pulp and periapical diseases [1]. In the treatment, stem cells derived from tooth differentiate into various functional cells after activation and then form root and dental pulp-dentin complex-like structures [2]. Human dental pulp stem cells (hDPSCs), one of mesenchymal stem cells (MSCs) with high proliferation, self-renewal and multi-lineage differentiation potential, can be differentiated into odontoblasts and contribute to the formation of dentin and dental pulp-dentin complex [3]. Although a few mechanisms by which stem cells derived from tooth differentiate into odontoblasts have been discovered, it has not yet been fully revealed [4].

Non-coding RNA with a length greater than 200 nucleotides is called long non-coding RNA (lncRNA). lncRNA is a by-product of RNA polymerase II transcription. Its gene conservation is lower than mRNA encoding protein, but its promoter region and splicing site are highly conserved. Therefore, the expression of lncRNA at a specific time and location is of great importance [5, 6]. The small endogenous non-coding RNA single-stranded molecule with a length of usually 20–24 nucleotides is called microRNA (miRNA), which is mainly observed in the cytoplasm and almost completely regulates protein expression by interacting with messenger RNA (mRNA) [7]. lncRNA located in the cytoplasm can base-pair with miRNA and effectively consume them, thereby affecting mRNA expression [8].

Small nucleolar RNA host genes (SNHGs) are a category of long non-coding RNAs found in the nucleus and cytoplasm that regulate gene expression at several levels, including transcription, alternative splicing, and post-translational regulation [9]. According to reports, small nucleolar RNA host gene 1 (SNHG1) can affect the development of osteosarcoma by regulating miR-101-3p, miR-326, miR-577 [10–12] and can influence the proliferation of colorectal cancer cells via miR-154-5p [13]. These studies indicate that SNHG1 can exert its function by competitively binding to miRNAs. At the same time, SNHG1 can regulate the osteogenic differentiation of bone marrow mesenchymal stem cells (BMMSCs) and periodontal ligament stem cells (PDLSCs), respectively [14–16]. Furthermore, up-regulation of SNHG1 is detected during the differentiation of human dental pulp cells into odontoblast-like cells [17]. We can hypothesize that SNHG1 may affect the odontogenic differentiation of hDPSCs.

Here, the impact of SNHG1 on odontogenic differentiation of hDPSCs is discussed, as well as a part of its mechanism as a competitive endogenous RNA (ceRNA). Our findings reveal that SNHG1 can boost the odontogenic differentiation ability of hDPSCs by inhibiting the Wnt/β-catenin pathway through miR-328-3p.

## Methods

### Cell culture

The Ethics Committee of Nanjing Medical University School of Stomatology gave clearance to this study. After obtaining the patient’s informed consent in the Department of Oral Surgery of Jiangsu Provincial Stomatological Hospital, we collected orthodontic reduction premolars from healthy donors under 16 years old. The pulp tissue was carefully isolated from immature roots, rinsed in phosphate-buffered saline (PBS; Gibco, Life Technologies, USA), sliced, and then incubated at 37 °C in a 5% CO_2_ incubator with 3 mg/ml collagenase type I (Sigma, St. Louis, MO, USA). The tissue block and separated cells were collected after 30 min of digestion by centrifugation at 1000 r/min for 5 min. They were inoculated at 37 °C in a 5% CO_2_ incubator with the complete alpha minimum essential medium (α-MEM; Gibco, Life Technologies, USA) containing 100 U/mL penicillin and 100 mg/mL streptomycin (Gibco, Life Technologies, USA), and 10% fetal bovine serum (FBS; ScienCell Research Laboratories, California, USA). Every three days, the medium was changed. When the cells have reached 70–80% confluence, they are trypsin digested and passaged at a 1:3 ratio. Cells from stage 3 to passage 5 were utilized in the subsequent experiments.

### Flow cytometry

In order to identify the phenotype of hDPSCs, flow cytometry was applied to detect MSCs’ surface markers. According to the instructions, the cells at passage 3 were incubated with anti-human CD90, CD73, CD29, CD34, and CD45 fluorescent antibodies (BD Pharmingen, San Diego, CA, USA).

### Alkaline phosphatase (ALP) activity and staining

Transfected hDPSCs were planted in a 12-well plate and grown for 7 days in mineralization induction medium. The quantitative analysis of ALP activity was performed using an alkaline phosphatase detection kit (Jiancheng, Nanjing, China) in accordance with the instructions. After 30 min of fixation with 4% paraformaldehyde, ALP staining was conducted with the BCIP/NBT staining kit (Beyotime, Shanghai, China).

### Alizarin red S staining (ARS) and quantification

The transfected hDPSCs grown for 14 days in mineralization induction media, with the medium changing every three days. Before being stained with Alizarin Red S (Leagene Biotechnology, Beijing, China), the cells were fixed in 4% paraformaldehyde. The mineralized nodules were seen and photographed using an inverted microscope. The staining profile was scanned using the scanner. To quantify ARS, 10 percent cetylpyridinium chloride was utilized, and the OD value was measured at 562 nm absorbance. Calcium levels were normalized to total protein levels in each group.

### Stem cell multidirectional differentiation

hDPSCs were inoculated onto 6-well plates and grown in full medium to 80 percent confluence and above for odontogenic and adipogenic differentiation. 2 weeks after induction with odontogenic differentiation induction medium (10 mM β-glycerophosphate (Sigma, St. Louis, MO, USA), 50 μg/mL ascorbic acid (Sigma, St. Louis, MO, USA) and 10 nM dexamethasone (Sigma, St. Louis, MO, United States) diluted in 10% FBS α-MEM), the cells were identified with ARS. After 21–30 days of cell culture using OriCell® hDPSCs Adipogenic Differentiation Kit (Cyagen Biosciences, Guangzhou, China), Oil Red O staining was for cell identification. For chondrogenic differentiation, the right amounts of cells were cultured in a 15 ml conical centrifuge tube with OriCell® Human BMMSCs Chondrogenic Differentiation Kit (Cyagen Biosciences, Guangzhou, China) for 1–2 days to form cartilage ball. The cartilage ball was carefully inoculated into a 48-well plate for 21 days after induction, then fixed with 4% paraformaldehyde, frozen sectioned and identified by Alcian blue staining.

### Quantitative reverse transcription -polymerase chain reaction (qRT-PCR)

Total cell RNA was extracted with Trizol reagent (Invitrogen, New York, NY, USA), and concentration quantification and purity detection were performed with a Nanodrop spectrophotometer. Then HiScript II Q RT SuperMix for qPCR (Vazyme, Nanjing, China) was used to synthesize cDNA. Finally, ChamQ Universal SYBR qPCR Master Mix (Vazyme, Nanjing, China) was used for quantification. Table 1 lists the primer sequences that were employed. For miRNA, cDNA was reverse transcribed by stem-loop primers and miRNA 1st Strand cDNA Synthesis Kit (Vazyme, Nanjing, China). *U6* was used for miRNA standardization, whereas *GAPDH* was utilized to normalize mRNA and lncRNA expression. The relative expression levels were determined using the 2^−ΔΔCT^ method. In comparison with the control condition, the findings were reported as fold changes.

**Table 1:** Primer sequences

Genes	Primes	Sequences (5′–3′)
*GAPDH*	Forward	GGAGCGAGATCCCTCCAAAAT
	Reverse	GGCTGTTGTCATACTTCTCATGG
*SNHG1*	Forward	GGAACAGACACGAAGTGGA
	Reverse	AGAACCCAAACTCAGGCA
*ALP*	Forward	GAGATGTTGTCCTGACACTTGTG
	Reverse	AGGCTTCCTCCTTGTTGGGT
*DMP-1*	Forward	AGGAAGTCTCGCATCTCAGAG
	Reverse	TGGAGTTGCTGTTTTCTGTAGAG
*DSPP*	Forward	ATATTGAGGGCTGGAATGGGGA
	Reverse	TTTGTGGCTCCAGCATTGTCA
*U6*	Forward	CTCGCTTCGGCAGCACA
	Reverse	AACGCTTCACGAATTTGCGT
*miR-328-3p*	Forward	GCTGGCCCTCTCTGCCC
	Reverse	AGTGCAGGGTCCGAGGTATT
*Axin1*	Forward	GACCTGGGGTATGAGCCTGA
	Reverse	GGCTTATCCCATCTTGGTCATC

### Cell transfection

SNHG1 overexpressed virus and specific siRNA, has-miR-328-3p mimics and inhibitor are all prepared by GenePharma (Shanghai, China) (Sequences in Additional file 1). hDPSCs were inoculated overnight and infected with lentivirus in the presence of polybrene (5 μg/mL) for 16–24 h. siRNA, mimics and inhibitors were transiently transfected with Lipofectamine 2000 (Invitrogen, New York, NY, USA) in serum-free medium for 6 h and then replaced with complete medium.

### Cell counting kit-8 (CCK-8) assay

The proliferation activity of hDPSCs was detected with CCK-8 (Dojindo, Tokyo, Japan). The transfected cells were planted at a density of 2000 cells per well on a 96-well culture plate. At particular time points (day 0, 1, 3, 5 and 7), 1:9 ratios of CCK-8 reagent and α-MEM were injected into each well. OD value was determined using a microplate reader at 450 nm absorbance after 2 h of incubation.

### Western blot

The total protein was extracted from the cells by protein lysate. On 10% SDS-PAGE gels, equal quantities of proteins were isolated and then transferred to 0.22 m polyvinylidene fluoride membranes (Millipore Corporation, Billerica, MA, USA). After being blocked with 5% skim milk for 1 h, the membranes were incubated with the following antibodies overnight at 4 °C: GAPDH (Proteintech, 10,494–1-AP), DSPP (Bioword, BS71212), DMP-1 (Affinity, DF8825, RRID: AB_2842022), ALP (Abcam, ab95462), Axin1 (Cell Signaling Technology, #2087), β-catenin (Cell Signaling Technology, #8480), Active β-catenin (Cell Signaling Technology, #19,807). The membranes were incubated secondary antibody for 1 h at room temperature and then exposed using the Immobilon Western Chemiluminescent HRP substrate (Millipore Corporation, Billerica, MA, USA). The protein bands were subsequently quantified with Image J software.

### Immunofluorescence staining

The transfected hDPSCs were grown to an adequate density on glass coverslips in 12-well plate. The cells were rinsed in PBS and fixed with 4% paraformaldehyde. After washing with PBS, the cells were perforated with 0.25% Triton-100 (Beyotime, Shanghai, China) for 12 min and blocked with goat serum at 4 °C overnight. Then, the coverslips were incubated with DSPP and DMP-1 primary antibodies at 4 °C overnight. The coverslips were overlaid with the fluorescent dye-labeled secondary antibody and kept in dark at room temperature for 1 h. Finally, the glass coverslips were covered with the anti-fluorescence quencher agent containing 4′,6-diamidino-2-phenylindole (DAPI; Beyotime, Shanghai, China) and then photographed under an inverted fluorescence microscope. ImageJ software was used for semi-quantitative analysis.

### Dual-Luciferase reporter assay

293 T cells were seeded to 70 percent to 80 percent confluence in a 24-well plate. Wild-type or mutant SNHG1 luciferase plasmids, as well as negative control or miR-328-3p mimics, were co-transfected into 293 T. Luciferase activity of Renilla and firefly was measured 24 h after transfection, depending on Dual-Luciferase Reporter Assay System (Promega, Madison, United States). The light intensity from firefly luciferase is normalized by Renilla luciferase.

### Statistical analysis

Data from three independent experiments were analyzed by GraphPad Prism software (version 8.0; GraphPad Software, San Diego, CA) and represented the mean and standard deviation (mean ± SD). The Social Science Statistical Package (SPSS) software (version 26.0) was used for statistical analysis. To compare the differences between groups, the Student’s t test was implemented. *P* < 0.05 indicates statistical significance.

## Results

### Characterization of hDPSCs

**Fig. 1:**
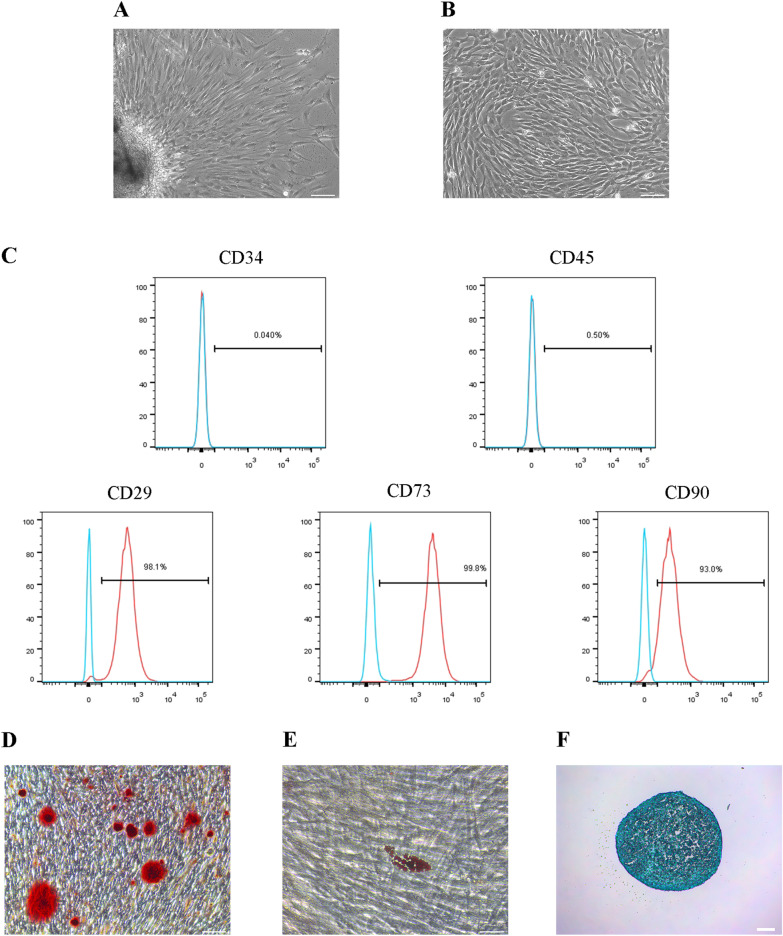
Cultivation and characterization of hDPSCs. **A** Primary hDPSCs at day 3. Scale bar = 100 μm. **B** hDPSCs at passage 3. Scale bar = 100 μm. **C** Flow cytometry analysis showing negative expression of CD34 and CD45 and positive expression of CD29, CD73 and CD90 in hDPSCs. **D**-**F** Multiple differentiations of hDPSCs. Alizarin red staining assayed osteo/odontogenic differentiation. Scale bar = 100 μm. Oil red O staining assayed adipogenic differentiation. Scale bar = 50 μm. Alcian blue staining assayed chondrogenic differentiation. Scale bar = 100 μm

The hDPSCs were successfully isolated from the collected immature permanent teeth, which were adherent cells with a spindle-shaped morphology (Fig. 1A, B). The surface markers of the isolated hDPSCs were characterized using flow cytometry. The results revealed that these cells expressed CD29, CD73, and CD90 while negatively expressing CD34 and CD45 (Fig. 1C), indicating that they were MSCs. Alizarin red, oil red O, and Alcian blue staining were used to validate the isolated hDPSCs' capacity to differentiate into osteo/odontogenic, adipogenic, and chondrogenic cells, respectively (Fig. 1D–F).

### SNHG1 expression increases in hDPSCs during the odontogenic differentiation, and SNHG1 promotes odontogenic differentiation of hDPSCs

**Fig. 2:**
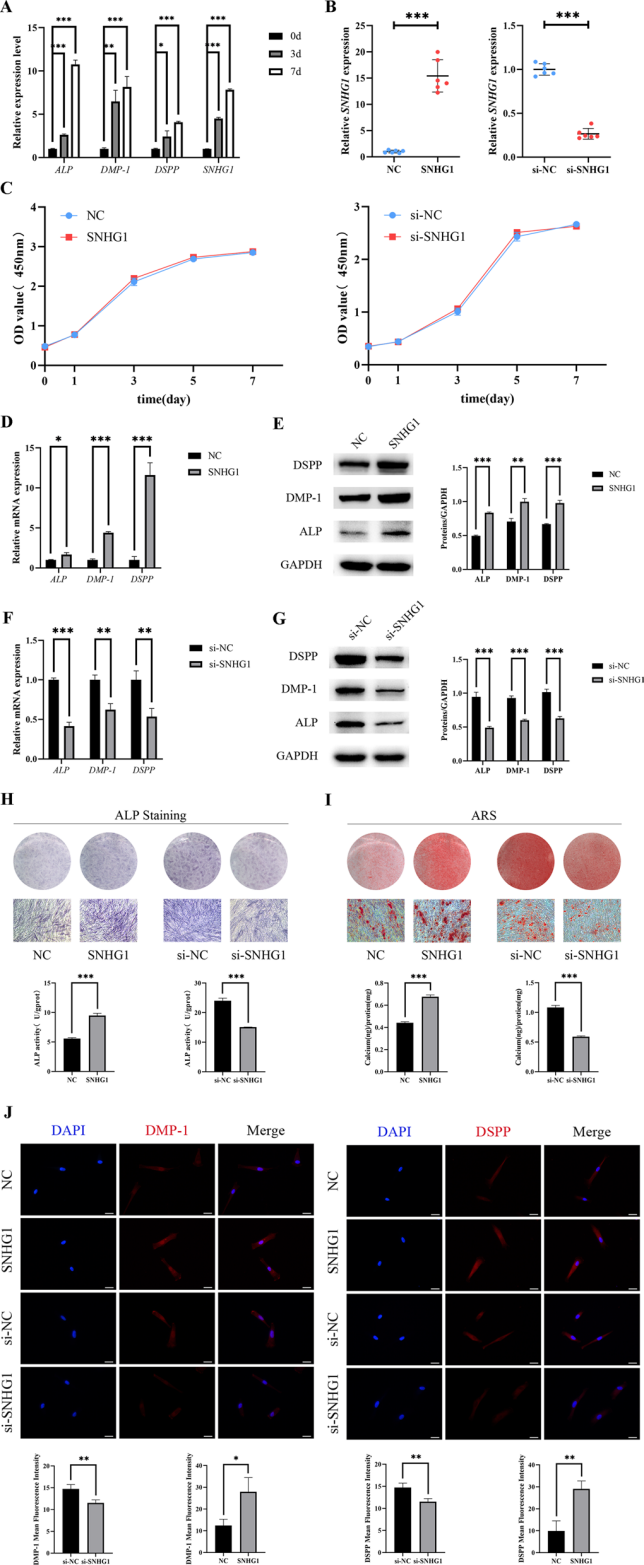
SNHG1 expression in hDPSCs during the odontogenic differentiation and SNHG1 governs the odontogenic differentiation of hDPSCs. **A** Relative *ALP, DMP-1, DSPP* and *SNHG1* expressions were measured by qRT-PCR at day 0, 3, and 7 under the mineralization induction condition. GAPDH was used for normalization. **B** The transfection efficacy of SNHG1 was measured by qRT-PCR in SNHG1 group and si-SNHG1 group, with GAPDH as a control. **C** The proliferation ability of hDPSCs was measured by CCK-8. **D**-**G** After SNHG1 overexpression or silence, relative *ALP, DMP-1* and *DSPP* expression were measured by qRT-PCR and ALP, DMP-1 and DSPP expression were measured by Western blot at day 5 under the mineralization induction condition. **H** At 7 days after odontogenic induction, hDPSCs were performed with ALP staining and activity. **I** On the 14th day, Alizarin Red staining and quantification were performed. **J** The expression of DMP-1 and DSPP in transfected hDPSCs were also showed by Immunofluorescence Staining. Scale bar = 20 μm. *P < 0.05, **P < 0.01, ***P < 0.001

To verify whether SNHG1 is involved in odontogenic differentiation, we detected the expression of SNHG1 and odontogenic differentiation marker genes (*dentin sialophosphoprotein* (*DSPP*), *dentine matrix protein 1* (*DMP-1*) and *ALP*) during the differentiation of hDPSCs into odontoblasts (Fig. 2A). The results showed that odontogenic induction elevated the expression of SNHG1 in hDPSCs considerably.

We performed cell transfection to construct SNHG1 silenced and overexpressed in hDPSCs to study the role of SNHG1 in odontogenic differentiation. qRT-PCR study validated the transfection effect (Fig. 2B). To begin, the CCK-8 assay revealed that SNHG1 overexpression had no effect on the proliferation of hDPSCs (Fig. 2C). There was the same result between si-NC group and si-SNHG1 group (Fig. 2C). The mRNA and protein expression of DSPP, DMP-1, and ALP in the SNHG1 group were considerably up-regulated after five days of culture using mineralization induction media, compared to the NC group (Fig. 2D, E). The similar trends were seen in ALP staining and activity, ARS and immunofluorescence staining (Fig. 2H–J). SNHG1 overexpression increased the odontogenic differentiation of hDPSCs, according to these findings. On the contrary, si-NC and si-SNHG1 groups demonstrated that silencing SNHG1 could inhibit the tendency (Fig. 2F–J). In a nutshell, SNHG1 promoted odontogenic differentiation of hDPSCs.

### SNHG1 can bind to miR-328-3p and affects its expression

**Fig. 3:**
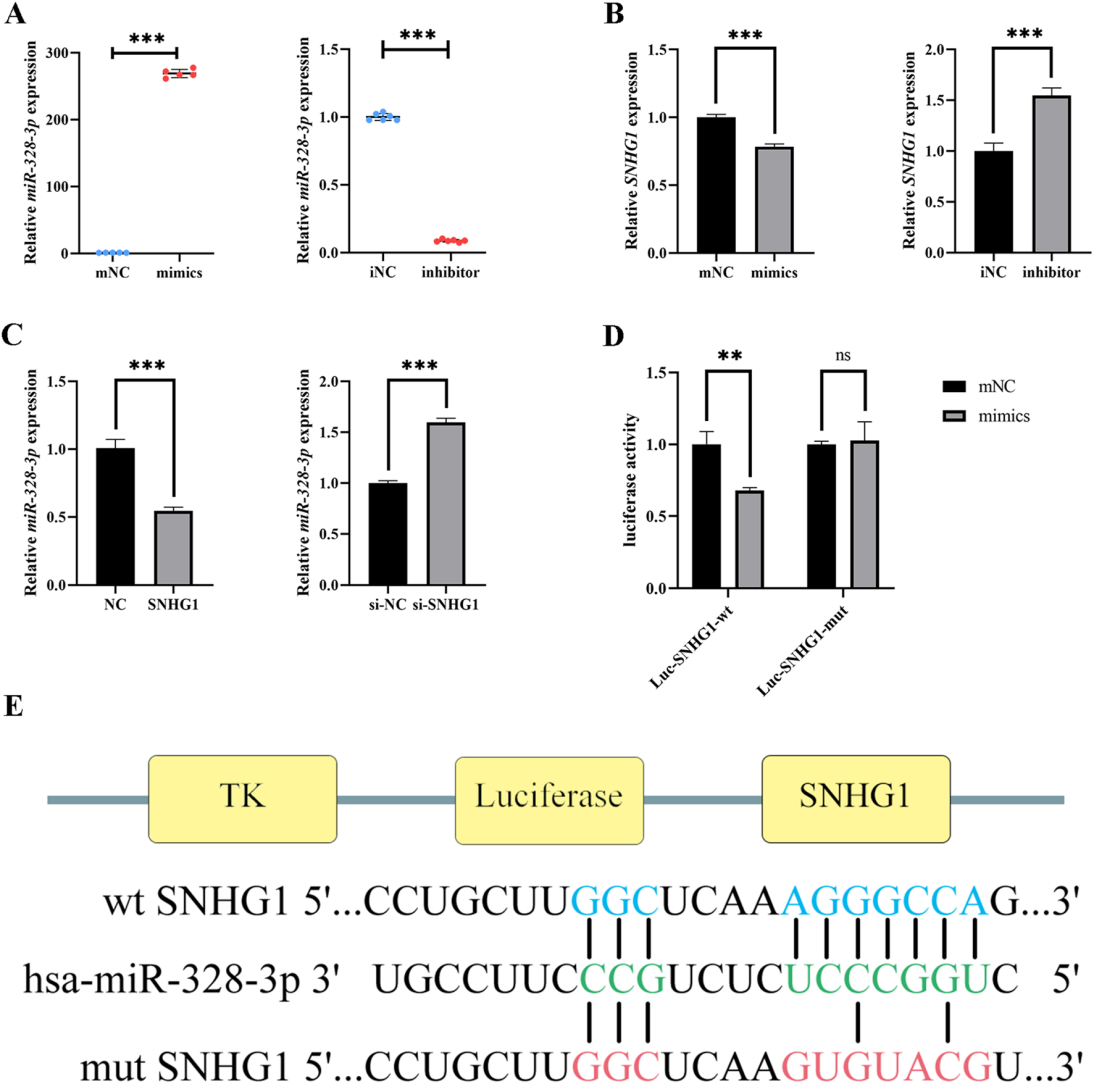
SNHG1 acts as a ceRNA for miR-328-3p. **A** The transfection efficacy of miR-328-3p mimics and inhibitor was verified by qRT-PCR. **B** The expression of SNHG1 was regulated by miR-328-3p. **C** SNHG1 can also govern the expression of miR-328-3p. **D** The dual-luciferase reporter assay was conducted in 293 T cells. The luciferase activities of the wild-type or Mutant SNHG1 reporter gene were detected after 24 h of co-transfection with mimics NC or miR-328-3p mimics. The light intensity from firefly luciferase is normalized by Renilla luciferase. **E** The potential binding sites of SNHG1 and miR-328-3p predicted by the biological prediction software and the site changes in mutant SNHG1. *P < 0.05, **P < 0.01, ***P < 0.001

SNHG1 has been found in the nucleus and cytoplasm, and it can regulate multiple biological processes via the ceRNA mechanism [13, 18]. Using LncBase Predicted v.2 in the DIANA tool and StarBase v2.0, we looked for prospective miRNAs targeting SNHG1 [19, 20]. Combined with the results retrieved from the literature that miR-328-3p has significant changes in osteogenic lineage differentiation [21, 22], it was speculated that miR-328-3p might target SNHG1 during odontogenic differentiation of hDPSCs. After miR-328-3p mimics and inhibitor were transfected into hDPSCs, we checked the transfection effectiveness of them (Fig. 3A). SNHG1 expression was suppressed by miR-328-3p (Fig. 3B). SNHG1 could potentially act as a negative regulator of miR-328-3p expression (Fig. 3C). The luciferase activity of the wild-type SNHG1 reporter gene was considerably decreased by miR-328-3p mimics, and the predicted binding site mutation successfully reversed the previous inhibition (Fig. 3D, E). The above results proved the direct binding between SNHG1 and miR-328-3p.

### miR-328-3p inhibits odontogenic differentiation of hDPSCs

**Fig. 4:**
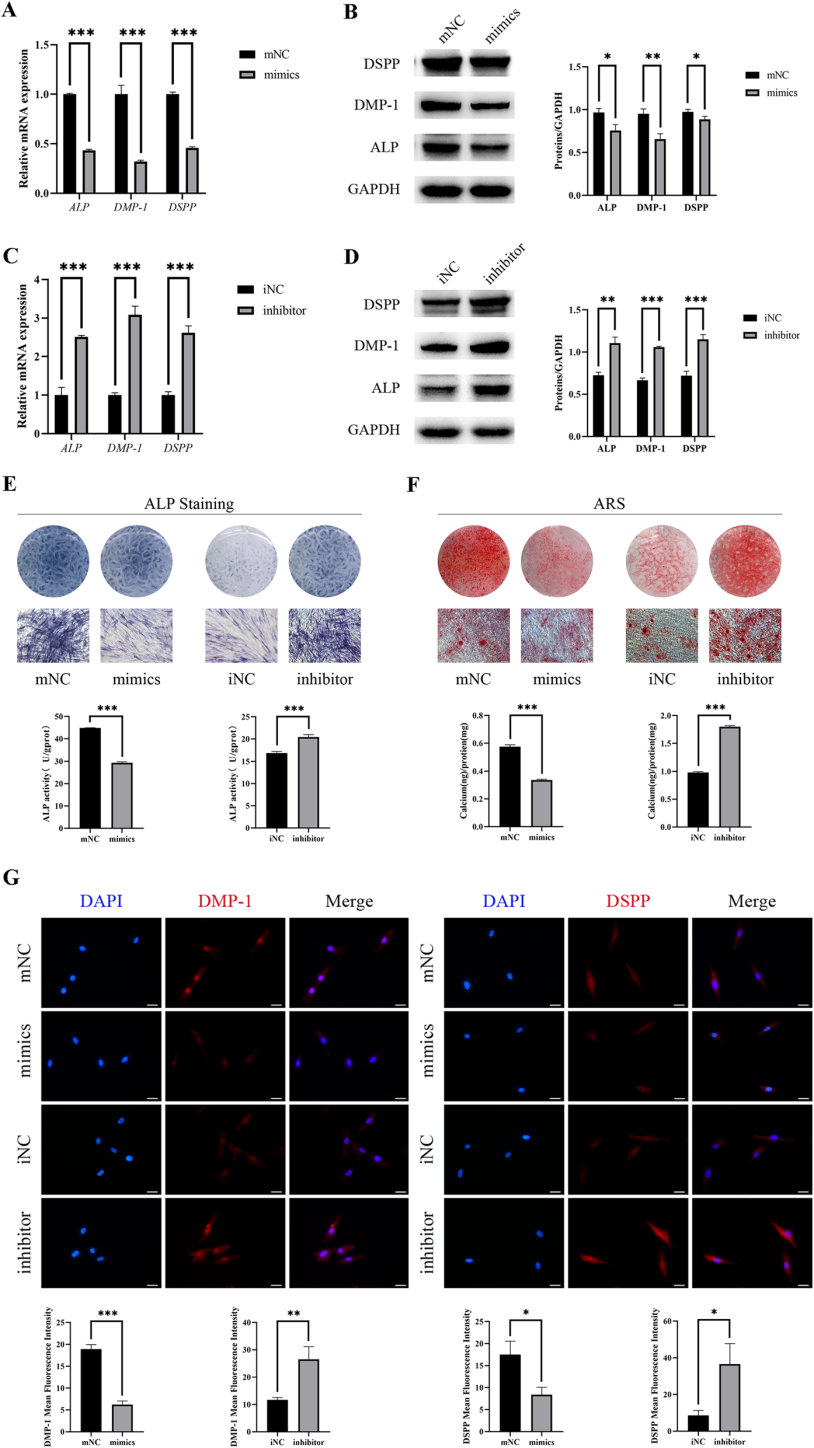
MiR-328-3p effects the odontogenic differentiation of hDPSCs. **A**-**D** After cell transfection of miR-328-3p mimics and inhibitor, relative *ALP, DMP-1* and *DSPP* expression and ALP, DMP-1 and DSPP expression were measured by qRT-PCR and Western blot on the 5th day of mineralization induction. **E**, **F** On the 7th day of mineralization induction, ALP staining and activity were performed to determine the odontogenic differentiation of hDPSCs. Similarly, Alizarin Red staining and quantification were performed on the 14th day. Scale bar = 100 μm. **G** Immunofluorescence Staining showed different expression levels of DMP-1 and DSPP in transfected hDPSCs. Scale bar = 20 μm. *P < 0.05, **P < 0.01, ***P < 0.001

miR-328-3p was overexpressed and knocked down utilizing miR-328-3p mimics and inhibitor. mNC, mimics, iNC, and inhibitor were the four groups of transfected cells. After five days of culture in the mineralization induction medium, overexpression of miR-328-3p inhibited the mRNA and protein expression of DSPP, DMP-1 and ALP, whereas downregulation of miR-328-3p boosted the expression of odontogenic differentiation marker genes (Fig. 4A–D). Immunofluorescence staining further confirmed that miR-328-3p mimics could hinder odontogenic differentiation, and miR-328-3p inhibitors can promote this process (Fig. 4G). In the mimics group, ALP activity, ALP staining, and ARS were all reduced, but in the inhibitor group, they were all increased(Fig. 4E, F). In summary, miR-328-3p inhibited odontogenic differentiation of hDPSCs.

### miR-328-3p partially mediates SNHG1's pro-odontogenic action

**Fig. 5:**
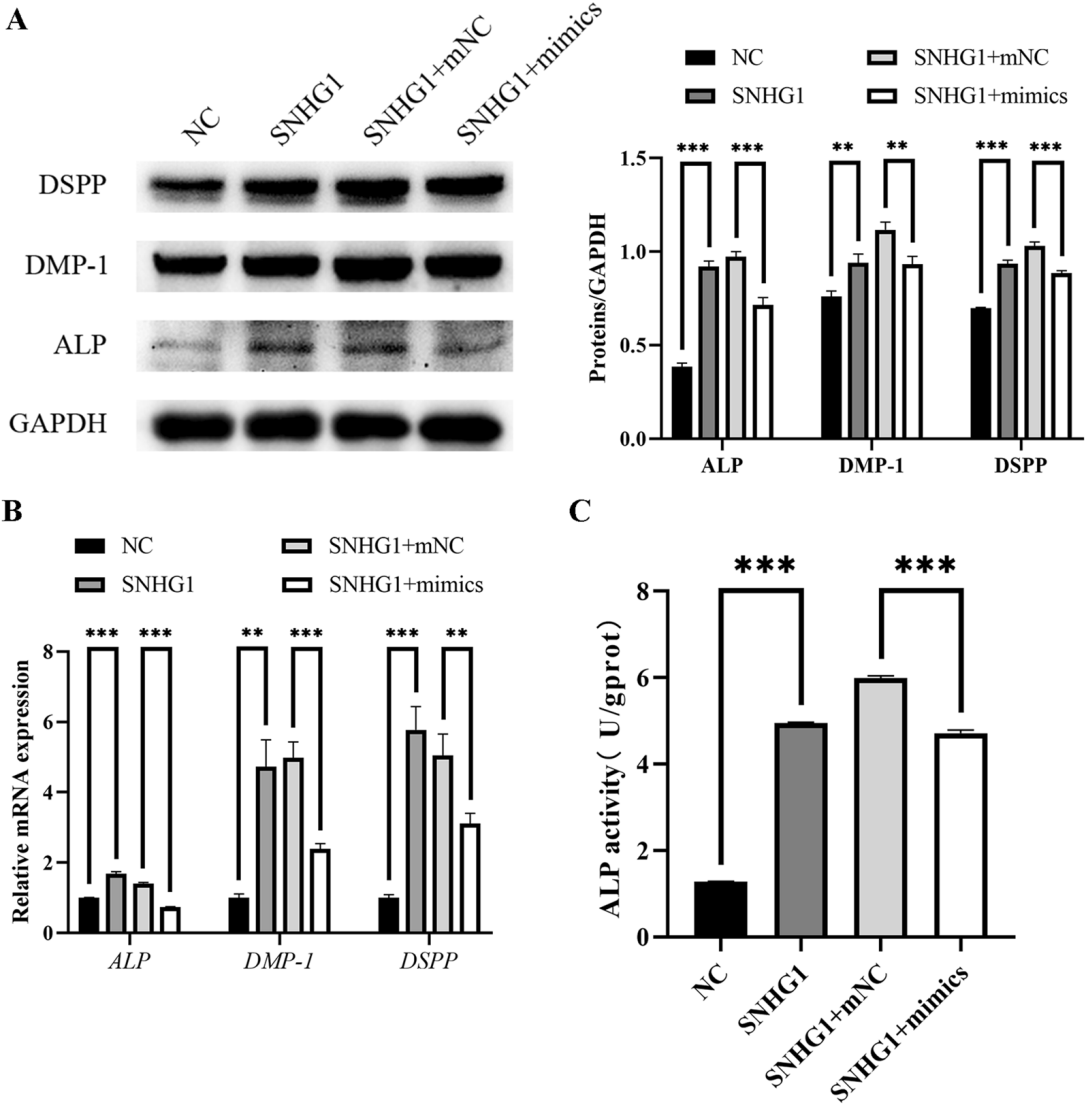
miR-328-3p partially mediates the pro-odontogenic function of SNHG1. **A**, **B** Exogenous miR-328-3p partially rescued the mRNA and protein expression of ALP, DMP-1 and DSPP in the hDPSCs overexpressing SNHG1. **C** miR-328-3p also rescued the up-regulation of ALP activity caused by SNHG1 overexpression. *P < 0.05, **P < 0.01, ***P < 0.001

We transfected miR-328-3p mimics into stably expressing SNHG1 hDPSCs and cultivated them with mineralization induction medium to determine whether the pro-odontogenic effect of SNHG1 was mediated by miR-328-3p. Exogenous miR-328-3p partially rescued the mRNA and protein expression of odontogenic differentiation marker genes in hDPSCs overexpressing SNHG1 (Fig. 5A, B). In addition, this tendency was also detected in ALP activity (Fig. 5C).

### SNHG1 inhibits the Wnt/β-catenin pathway via competitively binding miR-328-3p in the odontogenic differentiation of hDPSCs

**Fig. 6:**
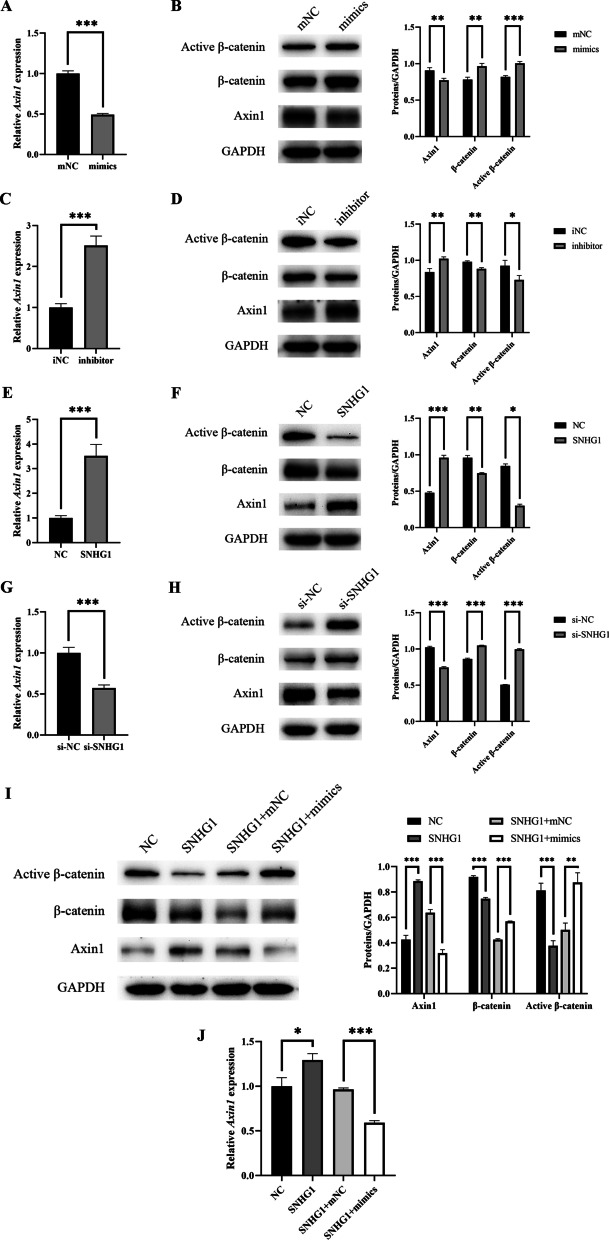
SNHG1 regulates Wnt/β-catenin pathway via competitively binding miR-328-3p in the odontogenic differentiation of hDPSCs. **A**-**D** After miR-328-3p was overexpressed, Axin1 was down-regulated at the gene and protein level, thereby promoting Wnt/β-catenin pathway, which manifests as an up-regulation of (active) β-catenin. After miR-328-3p was silent, there were opposite effects. **E**–**H** SNHG1 had opposite effects on *Axin1*, Axin1 and (active) β-catenin expression, compared with miR-328-3p. **I** Western blot analysis indicated that the miR-328-3p mimics rescued the SNHG1 mediated up-regulation of Axin1 and (active) β-catenin. **J** qPCR validated western blot results of Axin1 at the gene level. *P < 0.05, **P < 0.01, ***P < 0.001

It has been confirmed that miR-328-3p can regulate the Axin1/β-catenin cascade[23]. Therefore, we detected *Axin1* expression and Axin1/β-catenin cascade protein expression in hDPSCs during odontogenic differentiation. Down-regulated Axin1 and up-regulated β-catenin were discovered in mimics group (Fig. 6A, B), while the opposite in inhibitor group (Fig. 6C, D). These findings indicated that miR-328-3p inhibited Axin1 protein expression to up-regulate Wnt/β-catenin pathway. We hypothesized that SNHG1 would regulate Axin1 protein production via miR-328-3p, hence adjusting the Wnt/β-catenin pathway. To verify this, we detected *Axin1* expression and the protein levels of Axin1/β-catenin cascade in si-NC, si-SNHG1, NC, SNHG1, SNHG1 + mNC, and SNHG1 + mimics groups, respectively (Fig. 6E–J). The results demonstrated that overexpression of SNHG1 enhanced Axin1 mRNA and protein levels in hDPSCs, but the promotion effect was neutralized after co-transfection with miR-328-3p mimics. β-catenin protein expression was the opposite. In conclusion, SNHG1 is linked to Wnt/β-catenin pathway through miR-328-3p.

## Discussion

Regenerative endodontics uses the concept of tissue engineering to regenerate the pulp-dentin complex of immature permanent teeth with necrotic pulp due to infection, trauma or developmental abnormalities [24]. Stem cells are a critical factor in the regeneration of dental pulp tissue engineering [25]. Exploring the processes of hDPSCs' odontogenic differentiation would thus aid in the advancement of this technology. This study found that SNHG1 up-regulates Axin1 through miR-328-3p, thereby inhibiting Wnt/β-catenin pathway to promote the differentiation of hDPSCs.

Recent study has connected lncRNAs to the odontogenic differentiation of dental tissue-derived MSCs [4]. For instance, through epigenetic control of DLX3 by the H19/SAHH axis, lncRNA H19 regulates hDPSCs odontogenic differentiation [26]. lncRNA CALB2 is also a sponge for miR-30b-3p, which promotes odontogenic differentiation of hDPSCs by up-regulating RUNX2 [27]. SNHG1, which belongs to the non-coding RNA family that carries snoRNA [9], can control the osteogenic differentiation of MSCs [14–16], but its involvement in odontogenic differentiation is unknown.

The effect of SNHG1 on the proliferation and differentiation of hDPSCs was investigated in this study. Above our findings revealed that SNHG1 had no impact on the proliferation of hDPSCs. This is distinct from the research results related to cancer cells, revealing that the regulatory effect of SNHG1 on cell proliferation is complicated [10–13]. As shown in the results, the overexpression of SNHG1 positively regulates hDPSCs’ odontogenic differentiation, while SNHG1 negatively regulates MSCs’ osteogenic differentiation in other studies [14, 15]. On the one hand, this may be due to different cell lineages for experiments. On the other hand, although the mechanisms of osteogenic and odontogenic differentiation are similar, there are still certain differences [28].

SNHG1 is expressed in both the cytoplasm and the nucleus, according to several studies, indicating the variety of SNHG1 functions [9, 13, 29]. In the cytoplasm, SNHG1 can act as a microRNA sponge [30, 31]. Our evidence shows that miR-328-3p partially mediates the pro-odontogenic function of SNHG1. Only a tiny amount of literature previously stated that miR-328-3p could alter osteogenic differentiation in osteoporosis, and there are inadequate investigations on it in cell differentiation [21, 22]. An emerging study has confirmed that miR-328-3p can rescue damaged MSCs in osteogenesis [23].

Then we looked into the mechanism by which miR-328-3p controls hDPSCs’ odontogenic differentiation. We searched related research and discovered that Axin1 is regulated by miR-328-3p in mouse MSCs, and it also participates in and negatively regulates the canonical Wnt/β-catenin pathway [32–34]. Wnt/β-catenin signaling pathway is important for MSCs’ differentiation [35]. We speculated that SNHG1 could bind miR-328-3p to inhibit the Wnt/β-catenin pathway, thereby promoting the odontogenic differentiation of hDPSCs. The results confirmed that overexpression of miR-328-3p could activate the Wnt/β-catenin pathway, and SNHG1 combined with miR-328-3p attenuated the promotion of the Wnt/β-catenin pathway. However, the effect of Wnt/β-catenin signaling pathway on the odontogenic differentiation of hDPSCs has different opinions in the existing studies [17, 36]. This inconsistency may be linked to Wnt activity level, cell source and experimental conditions. Furthermore, the actual mechanism of miR-328-3p acting on Wnt/β-catenin and how Wnt/β-catenin functions in odontogenic differentiation of hDPSCs remain uncertain that have to be researched further. The canonical Wnt/β-catenin signal has been demonstrated to boost osteogenic differentiation of MSCs by directly stimulating the expression of *Runx2* [37]. Runx2 inhibits the terminal differentiation of odontoblasts [38, 39]. Therefore, we can speculate that Wnt/β-catenin pathway may directly stimulate *Runx2* expression to inhibit the terminal odontogenic differentiation of hDPSCs, which needs experiments to prove.

In general, highly simulated in vivo animal experiments are required to reflect the therapeutic implications of basic research. At present, according to the formation position of the dental pulp tissue, the animal models of regenerative dental pulp therapy can be divided into: ectopic, semiorthotopic, and orthotopic[40]. Among them, the ectopic and semiorthotopic regeneration models of mice may be less suitable for our experiments, as the presence of mouse blood and cells in the model would interfere with the study on the mechanism between miR-328-3p and Wnt/β-catenin [41]. The orthotopic regeneration models can better simulate the conditions of regenerative endodontic procedures, and there is no problem of mouse gene interference, which is conducive to the subsequent in vivo experiments [41–43].

## Conclusion

In short, lncRNA SNHG1 inhibits the Wnt/β-catenin pathway through miR-328-3p and then promotes the odontogenic differentiation of hDPSCs. Our findings provide a theoretical foundation for the practical use of REPs.

## Supplementary Information

**Additional file 1**: The sequences of siRNA, mimics and inhibitor

## Data Availability

The datasets used and analyzed during the current study are available from the corresponding author on reasonable request.

## Abbreviations

REPs: Regenerative endodontic procedures
hDPSCs: Human dental pulp stem cells
MSCs: Mesenchymal stem cells
lncRNA: Long non-coding RNA
miRNA: MicroRNA
mRNA: Messenger RNA
SNHGs: Small nucleolar RNA host genes
SNHG1: Small nucleolar RNA host gene 1
BMMSCs: Bone marrow mesenchymal stem cells
PDLSCs: Periodontal ligament stem cells
ceRNA: Competitive endogenous RNA
PBS: Phosphate-buffered saline
α-MEM: Alpha minimum essential medium
FBS: Fetal bovine serum
qRT-PCR: Quantitative real-time-polymerase chain reaction
CCK-8: Cell Counting Kit-8
WB: Western Blot
DAPI: 4′,6-Diamidino-2-phenylindole
ALP: Alkaline phosphatase
ARS: Alizarin Red S staining
DSPP: Dentin sialophosphoprotein
DMP-1: Dentine matrix protein 1
