# Supplementary material for: lncRNA SNHG1 regulates odontogenic differentiation of human dental pulp stem cells via miR-328-3p/Wnt/β-catenin pathway
Source: Stem Cell Res Ther. 2022 Jul 15;13:311. doi: 10.1186/s13287-022-02979-w (PMC9284872; doi:10.1186/s13287-022-02979-w)
Supplement: Supplementary file 1 — Additional file 1: The sequences of siRNA, mimics and inhibitor [file 13287_2022_2979_MOESM1_ESM.docx]

|  | Sequence (5'-3') |
| --- | --- |
| si-SNHG1 | CCAGCACCUUCUCUCUAAATT |
|  | UUUAGAGAGAAGGUGCUGGTT |
| mimics | CUGGCCCUCUCUGCCCUUCCGU |
|  | GGAAGGGCAGAGAGGGCCAGUU |
| inhibitor | ACGGAAGGGCAGAGAGGGCCAG |
